# Talus Idiopathic Versus Stress-Injury-Related Osteonecrosis and Its Resolution: A Case Report

**DOI:** 10.7759/cureus.50360

**Published:** 2023-12-11

**Authors:** Francisco Rodriguez Fontan, Kenneth J Hunt

**Affiliations:** 1 Department of Orthopedics, University of Colorado Anschutz Medical Campus, Aurora, USA

**Keywords:** fracture, stress injury, shock wave therapy, bone stimulator, osteonecrosis, avascular necrosis, talus, mri, female

## Abstract

A 34-year-old healthy long-distance runner sustained a possible stress injury to the talus. This injury progressed into osteonecrosis (ON) or might have presented idiopathically. This patient had a complete normal metabolic workup. Non-surgical management, including resting, activity modification, and bone stimulators, led to resolution. Serial exams and magnetic resonance imaging demonstrated gradual resolution of the ON. At two years old, she was pain-free and had returned to running. Talus ON is uncommon and even more so in the absence of metabolic disorders or precipitating trauma. This case presents a debatable stress injury, an overuse injury, or even an idiopathic ON. It healed with non-surgical management. Serial, advanced imaging surveillance was implemented. There is a lack of impactful literature regarding the management of early ON and a paucity of strong recommendations to guide non-surgical treatment options in the early stages. This presentation is quite debatable as to whether there was a stress fracture leading to ON or if it was idiopathic ON. Yet, these entities could easily overlap, and physicians and orthopedists should be aware.

## Introduction

Osteonecrosis (ON) of the talus is a rare entity. The ankle ON has been reported to account for 3-4% of ON affecting major joints (i.e., shoulder, hip, knee, and ankle). The traumatic versus atraumatic pathogenesis ratio has been reported to be 3 to 1 [1]. The talus is about 60% articular surface with no muscular nor tendinous attachments and depends on three nutritious vessels (primarily the artery of the sinus tarsi and sinus tarsi). The dome is the zone with the poorest vascular supply [1]. The degree of fracture displacement correlates with the risk of developing ON, and underlying medical conditions can negatively contribute to the ON cascade [2]. Management can be challenging, and there are a myriad of non-surgical and surgical options where the goal is to stop the progression. In the setting of a displaced fracture, the indication is surgical fixation, but when atraumatic (i.e., alcoholism, corticosteroid use, rheumatological disease, or blood disorder) or idiopathic ON happens, the approach is different and may depend on the ON stage [2,3]. In a stepwise progression, non-surgical management for early stages involves protected weight bearing, activity modification, analgesics, and bracing, whereas in later stages, surgical management spans from core decompression and bone grafting towards tibiotalocalcaneal arthrodesis or total ankle versus talus replacement [2,4]. This case report presents a healthy young female with no underlying medical comorbidities who sustained an apparent unusual stress fracture to the talar neck believed to be secondary to microtrauma, which progressed into ON, or possible idiopathic ON and was successfully managed non-surgically. This case should alert physicians to a rare injury in long-distance runners and consider being vigilant of ON. Despite being an extraordinary presentation, its progression could be devastating to an athlete. Institutional review board exemption was obtained, and the patient was informed that data concerning the case would be submitted for publication and provided consent.

## Case presentation

**Figure 1:**
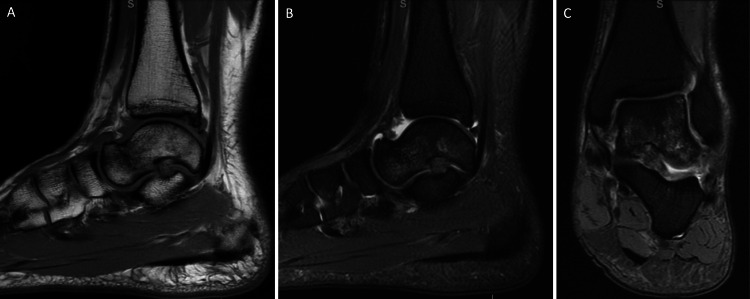
MRI of the left ankle - September 12, 2020. (A) T1-sequence, sagittal view shows a black irregular line at the talar neck demonstrating a possible stress fracture; (B-C) T2-sequence, sagittal and coronal views show signs of bone marrow hyperintensity demonstrating bone marrow edema. The posterior tibial plafond demonstrates a small osteochondral defect, with mild bone marrow edema.

**Figure 2:**
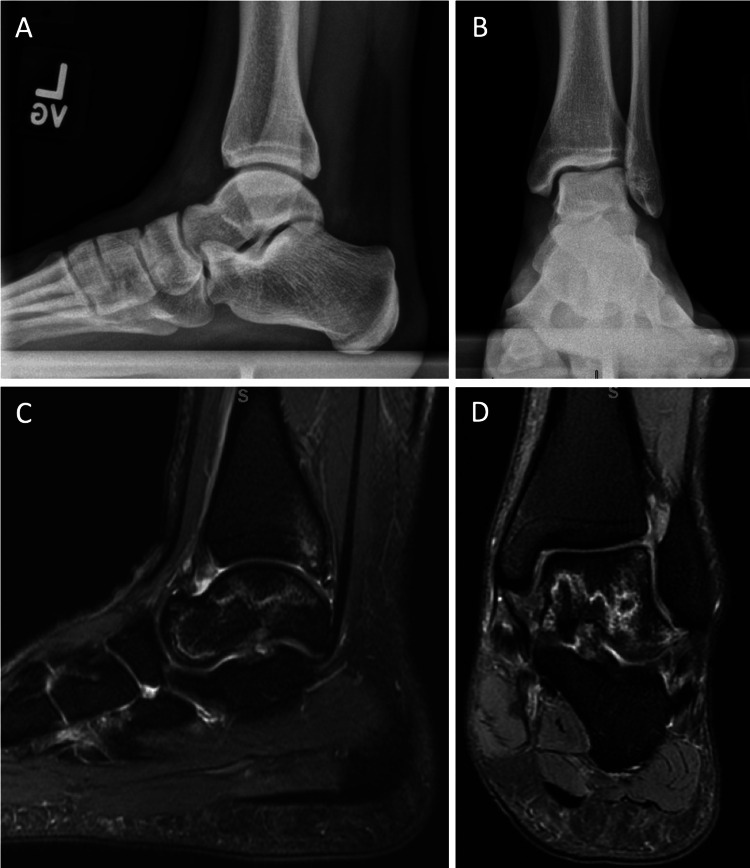
Radiographs and MRI of the left ankle at six months since onset of pain - March 31, 2021. (A-B) Lateral and anteroposterior X-rays demonstrating no acute injury, no signs of peritalar joint degeneration, or sclerosis. (C-D) T2-sequence, sagittal, and coronal views show a hyperintensity line, or “double line sign,” within the ischemic bone marrow and no subchondral collapse. The posterior tibial plafond re-demonstrates a small osteochondral defect, unchanged from the prior exam.

This is a healthy 34-year-old female long-distance runner with a body mass index in the normal range who presented to our clinic with an estimated six-month history of atraumatic chronic left ankle pain since November 2020. Prior to the onset of pain, she was typically running 30 miles a week with no significant change in her training regimen and had completed several marathons. She had no prior ankle injuries. At an outside hospital, they initially observed a stress injury to the talus neck on an MRI (Figure 1). The healthcare provider encouraged her to temporarily discontinue running and immobilized her in a boot. She had no pertinent past medical history for ON: alcoholism, steroid use, sickle cell disease, thalassemia, or rheumatologic disease, among others [1]. At our visit, she was seeking a second opinion. She was now six months since the onset of symptoms and stated she was still having pain despite discontinuing impact activities. Although she was able to do daily activities, she was unable to go back to running. On our assessment, she complained of deep ankle pain, a stable ankle, and focal tenderness to deep palpation on the medial and lateral aspects of the talus. Laboratory results were normal for vitamin D and calcium. A new MRI demonstrated ON of the talus (i.e., stage I of the Ficat and Arlet classification) and an associated stable-appearing small posterior tibial plafond osteochondral defect (Figure 2) [3]. In the absence of a loose fragment and minimal bone marrow edema, we decided to monitor over time and focus treatment management on the talus ON. She was encouraged to withhold from running and impact activities for the next three months, start using a bone stimulator twice a day (Exogen Bone Stimulator; Lavallette, NJ, USA), use rocker bottom shoes, and attend formal guided physical therapy sessions.

**Figure 3:**
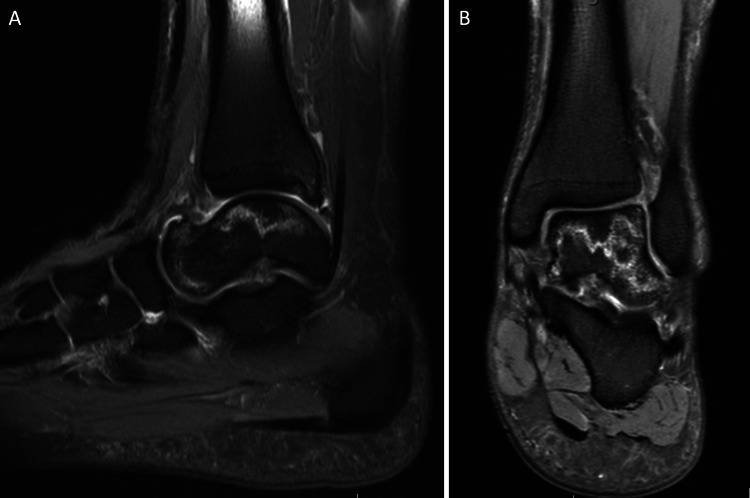
MRI of the left ankle at nine months - June 29, 2021. (A-B) T2-sequence, sagittal, and coronal views show a hyperintensity line with similar signs of osteonecrosis and preserved articular surface. The posterior tibial plafond re-demonstrates a stable small osteochondral defect.

At nine months from the onset of pain, she reported no pain with walking. She had not returned to running. On the exam, she continued to have minimal tenderness over the medial and lateral aspects of the talar neck. An MRI at that time showed ON within the talus in a similar location and similar intensity without evidence of collapse (Figure 3). The plan at this time was to do activity increments in an “impact ladder” way as symptoms dictate, beginning with comfort shoes and walking activities only and then gradually incorporating more activities.

**Figure 4:**
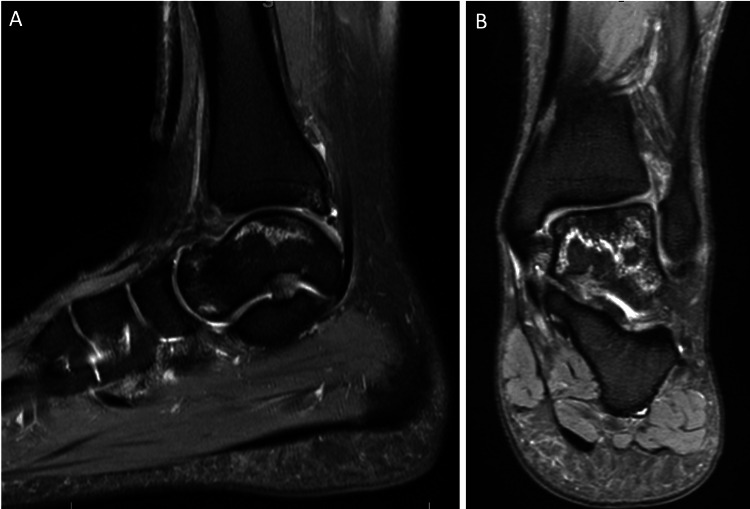
MRI of the left ankle at 12 months from injury - October 4, 2021. (A-B) T2-sequence, sagittal, and coronal views show ischemic bone marrow in a similar pattern with marginal improvement. The posterior tibial plafond re-demonstrates a stable small osteochondral defect.

**Figure 5:**
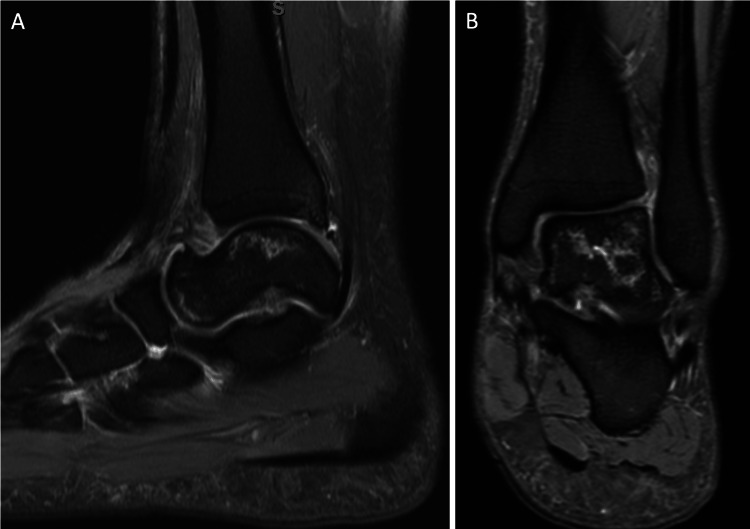
MRI of the left ankle at 15 months from injury - January 31, 2022. (A-B) T2-sequence, sagittal and coronal views show signs of osteonecrosis resolution with no collapse. The posterior tibial plafond re-demonstrates a stable small osteochondral defect.

At 12 months from the onset of symptoms, she was pain-free and had no tenderness throughout the ankle. An MRI at that time showed marginal improvement of the ON but no collapse (Figure 4). At this point, she was encouraged to gradually begin running. At 15 months, she had returned to running and had no pain, except for some achiness at night and minimal tenderness at the talus. MRI demonstrated improved signs of ON and no collapse (Figure 5). She was encouraged to continue running in gradual increments as symptoms dictated and to continue using the bone stimulator.

**Figure 6:**
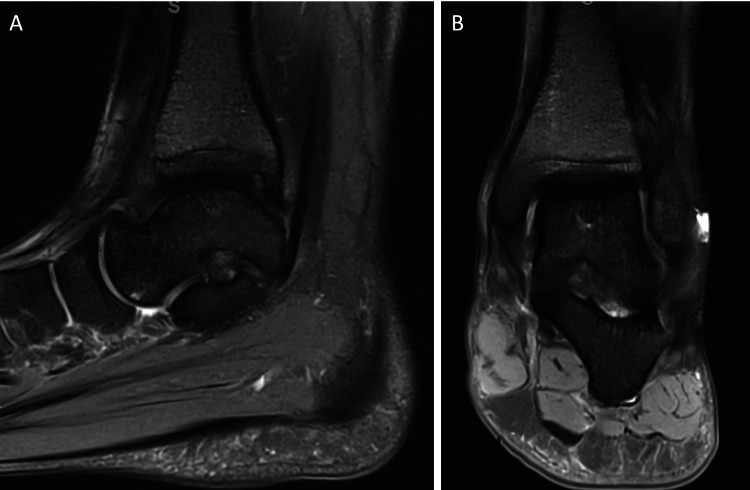
MRI of the left ankle at 2.5 years from injury - April 15, 2023. (A-B) T2-sequence, sagittal, and coronal views show significant improvement of the talar osteonecrosis with no “double line sign” and almost complete resolution. The posterior tibial plafond re-demonstrates a stable small osteochondral defect.

At 2.5 years from the onset of pain, by April 2023, she was pain-free for the last year and was back into running 30-50 miles a week. She had stopped using the bone stimulator for the last six months. MRI continued to show resolution of the talus ON and stable posterior tibial plafond small osteochondral defect (Figure 6). Her American Orthopedic Foot and Ankle Score (AOFAS) Ankle-Hindfoot scale was 100 [5].

## Discussion

This case report represents a successful non-surgically managed stage I talus ON from two possible entities: idiopathic or secondary to a stress injury [6]. Treatment involved physical therapy, activity modifications, and the use of a bone stimulator. The patient had no underlying precipitating medical condition to ON, except for a possible talus stress fracture that might have compromised the blood supply to the talus. A Hawkins type I talar neck fracture would be the most similar clinical circumstance to a stress fracture, as presented in this case report. Yet, the reported incidence of ON is below 13%, and these are secondary to high-energy trauma [7].

Although the stress fracture leading to ON was a possibility in the presented case, idiopathic ON could have been considered as well. Just as with idiopathic ON of the knee, this case presented with unilateral ON, and an MRI on presentation resembling early ON can be misinterpreted for a stress fracture (e.g., Figure 1) [8]. The “double line sign” representing the sclerotic rim of bone surrounded by hyperemic bone is not usually characteristic of idiopathic ON but rather a hypointense line resembling a stress fracture, as in Figure 1, which makes the stress fracture quite debatable [9]. Moreover, in subsequent MRIs, the classic pathognomonic “double line sign” was present throughout the talus [8]. Interestingly, one proposed theory of idiopathic ON is microtrauma, or an unrecognized traumatic event that could lead to subchondral microfracture, which certainly fits this case report [10]. This could have been a shear type of injury with rapid ON progression.

The patient showed clinical and radiological improvement over the course of 2.5 years, returning to the same level of activities and an "excellent" AOFAS score. In general, the stage of the disease and its extent can help guide treatment modalities. A meta-analysis by Dhillon et al. looked at outcomes from non-surgical and surgical treatment options and demonstrated five studies looking into non-surgical management for early post-traumatic ON of the talus [4]. Based on available “low quality” literature (i.e., level IV and retrospective observational studies), a period of non-weight bearing (e.g., nine months) with transition to gradual weight bearing with adjuvant patellar tendon bracing as symptoms dictate seems to be recommended [4,11]. About 50% of the time there is a reasonable clinical outcome, and one in three end up needing surgery [11].

Only one prospective study by Zhai et al. evaluated the use of extracorporeal shock wave therapy (i.e., bone stimulator modality) and found significant improvement in pain and necrosis at 18 months. They showed that AOFAS increased to 92.3 and the necrosis improved by 50% on MRI as opposed to standard physical therapy alone [12]. However, this modality warrants further investigation.

Another treatment option in early-stage talus ON, as with femoral head ON, is core decompression. It is a promising joint-preserving option for atraumatic talus ON. There are reported results of at least twofold improvement on Mazur and AOFAS, and 11% of the cases need arthrodesis [4,11]. Certainly, this option is doubtful of success in the setting of trauma and could have been performed in our case, but it was deferred as she continued to improve clinically and on interval MRIs. As for the case report presented in this article, given the patient’s improvement over time and being completely back to activities with no limitations, no further intervention or surveillance was warranted at the latest follow-up.

## Conclusions

To conclude, this is an unusually reported entity with a favorable outcome after non-surgical management. There is scarce literature regarding the management of early ON, as recent meta-analyses affirm the lack of strong recommendations to guide non-surgical treatment options in the early stages. The standard stepwise approach is to prevent progression and preserve the joint in the early stages and to perform arthrodesis or arthroplasty when collapse and joint degeneration happen. In addition, the presentation is quite debatable as to whether there was a stress fracture leading to ON or if it was idiopathic ON. Yet, these entities could easily overlap in this case. A low threshold for advanced imaging in highly active athletes with unusual presentations can guide further management in the early stages prior to radiographic changes.

## References

[1] Moon DK (2019). Epidemiology, cause, and anatomy of osteonecrosis of the foot and ankle. Foot Ankle Clin.

[2] Zhang H, Fletcher AN, Scott DJ, Nunley J (2022). Avascular osteonecrosis of the talus: current treatment strategies. Foot Ankle Int.

[3] Delanois RE, Mont MA, Yoon TR, Mizell M, Hungerford DS (1998). Atraumatic osteonecrosis of the talus. J Bone Joint Surg Am.

[4] Dhillon MS, Rana B, Panda I, Patel S, Kumar P (2018). Management options in avascular necrosis of talus. Indian J Orthop.

[5] Ibrahim T, Beiri A, Azzabi M, Best AJ, Taylor GJ, Menon DK (2007). Reliability and validity of the subjective component of the American Orthopaedic Foot and Ankle Society clinical rating scales. J Foot Ankle Surg.

[6] Chu EC, Bellin D (2015). Insufficiency fracture of the tibial plateau: a disease of rare diagnosis. J Osteopor Phys.

[7] Shamrock A, Byerly D (2022). Talar Neck Fractures. Talar Neck Fractures.

[8] Narváez J, Narváez JA, Rodriguez-Moreno J, Roig-Escofet D (2000). Osteonecrosis of the knee: differences among idiopathic and secondary types. Rheumatology (Oxford).

[9] Lee JK, Yao L (1988). Stress fractures: MR imaging. Radiology.

[10] Ecker ML, Lotke PA (1995). Osteonecrosis of the medial part of the tibial plateau. J Bone Joint Surg Am.

[11] Gross CE, Haughom B, Chahal J, Holmes GB Jr (2014). Treatments for avascular necrosis of the talus: a systematic review. Foot Ankle Spec.

[12] Zhai L, Zhang BQ, Wang JG, Xing GY (2010). Effect of liquid-electric extracorporeal shock wave on treating traumatic avascular necrosis of talus. J Clin Rehabil Tissue Eng Res.

